# Supplementary material for: A Computer-Assisted System for Early Mortality Risk Prediction in Patients with Traumatic Brain Injury Using Artificial Intelligence Algorithms in Emergency Room Triage
Source: Brain Sci. 2022 May 7;12(5):612. doi: 10.3390/brainsci12050612 (PMC9138998; doi:10.3390/brainsci12050612)
Supplement: Supplementary file 1 [file brainsci-12-00612-s001.zip › brainsci-1721001-supplementary.pdf]

Supplementary Table S1. Hyper-parameters range for experiments

Algorithm and Hyper-parameter	Values
Logistic regression	
penalty	l1, l2
C	np.logspace(-3,3,7), 1, 1.2, 5, 15, 30, 100
max_iter	100, 500, 1000, 2500
Random forest	
n_estimators	100, 500, 800, 1000
max_depth	15, 30, 50, 90, 150
min_samples_split	2, 4, 6, 10, 15
max_features	auto, 0.5, 1, 10, 30
class_weight	balanced, balanced_subsample
random_state	9, 15, 30, 45
SVM	
kernel	rbf, linear
C	1, 10, 100, 500, 1000, 1500, 3000
gamma	scale, 1e-2, 1e-3, 1e-4
LightGBM	
learning_rate	1e-3, 1e-2, 0.05, 0.1
num_iterations	100, 500, 800, 1000
max_depth	15, 30, 50, 90
random_state	9, 15, 30, 45
MLPClassifier	
hidden_layer_sizes	(200, 150, 75), (100,90,50), (100,70), (90,45)
earning_rate_init	1e-3, 1e-2
early_stopping	True, False
max_iter	100, 500
batch_size	auto, 16, 32, 50
XGBoost	
learning_rate	1e-4, 1e-3, 1e-2
gamma	1e-2, 1e-3, 1e-4, 1e-5
num_iterations	100, 500, 800
max_depth	15, 30, 50, 90
num_parallel_tree	1, 2, 5, 15
